# Conservative Treatment of Acute Colonic Diverticulitis

**DOI:** 10.1007/s11908-017-0600-y

**Published:** 2017-09-23

**Authors:** S. T. van Dijk, S. J. Rottier, A. A. W. van Geloven, M. A. Boermeester

**Affiliations:** 1 Department of Surgery, Academic Medical Center, Meibergdreef 9 1100 DD, PO Box 22660, Amsterdam, The Netherlands; 2 Department of Surgery, Tergooi Hospital, Hilversum, The Netherlands

**Keywords:** Acute diverticulitis, Conservative treatment, Uncomplicated, Antibiotics

## Abstract

**Purpose of Review:**

Since the treatment of acute diverticulitis has become more conservative over the last years, knowledge of conservative treatment strategies is increasingly important.

**Recent Findings:**

Several treatment strategies that previously have been imposed as routine treatment are now obsolete. Uncomplicated diverticulitis patients can be treated without antibiotics, without bed rest, and without dietary restrictions; and a selected group of patients can be treated as outpatients. Also, patients with isolated pericolic extraluminal air can be treated conservatively as well. Whereas some patient subgroups have been suggested to suffer from a more virulent disease course or higher recurrence rates, current evidence does not support all traditional understandings. Patients on immunosuppression or non-steroidal anti-inflammatory drugs seem to have a higher risk of complicated diverticulitis, but young patients do not. Data on the risk of recurrent diverticulitis in young patients is conflicting but the risk seems comparable to elderly patients. Besides the traditional treatments, several new treatment strategies have emerged but have failed thus far. Mesalazine does not have any beneficial effect on preventing recurrent diverticulitis based on current literature. Rifaximin and probiotics have been studied insufficiently in acute diverticulitis patients to conclude on their efficacy.

**Summary:**

This review provides an overview of recent developments in conservative treatment strategies of acute diverticulitis and discusses the latest evidence on patient subgroups that have been suggested to suffer from an aberrant disease course.

## Introduction

The incidence of diverticulosis and its complication acute diverticulitis is increasing worldwide and is imposing a growing burden on national healthcare systems [1]. In past years, there has been a strong tendency towards a more conservative treatment of acute diverticulitis, resulting in an expansion of knowledge about conservative treatment options and development of new treatment strategies [2]. This tendency is pointed out in the role of antibiotics in uncomplicated diverticulitis, outpatient rather than inpatient treatment, and pharmacological therapies that may replace surgery to prevent recurrent diverticulitis. Even for complicated diverticulitis, this same tendency of less aggressive treatment is observed with percutaneous abscess drainage or laparoscopic lavage for perforated diverticulitis. In addition, older treatment strategies like dietary restrictions and bed rest are still regularly used in daily practice. This review discusses the latest evidence on conservative treatment strategies for acute diverticulitis.

## Treatment of Acute Diverticulitis

### Diet

Dietary restrictions, from nil-per-mouth to liquids only or low-fiber diet, have been imposed as part of the routine treatment of acute diverticulitis. This is more tradition- than evidence-based. In recent years, two studies have shown that an unrestricted diet is not associated with an increase of diverticular complications [3, 4]. In a retrospective cohort study, no increase of diverticular perforation or abscess is found for solid food compared with a liquid diet, clear liquid diet, or nil per os [4]. Although patients with dietary restrictions tended to suffer from more severe diverticulitis (abscess) at presentation, results have not been adjusted for these confounders. Another, prospective single-arm cohort, study has included 86 uncomplicated diverticulitis patients; all with an unrestricted diet [3]. Eight per cent of these patients developed complications, including readmission for pain without diverticular complications, recurrent diverticulitis, surgery for ongoing symptoms, and free perforation. This rate is comparable to reported rates in literature. The quality of this evidence is very low, but conversely there is no evidence in favor of dietary restrictions. Therefore, an unrestricted diet seems justified in patients with acute diverticulitis, in the absence of planned invasive procedures that demand fasting.

### Bed Rest

Along with dietary restrictions, bed rest has been part of the routine treatment of acute diverticulitis. However, beneficial effects of bed rest have never been studied nor proven. Considering the overall trend of early mobilization in other medical research fields, of which beneficial effects have been proven in programs such as the Enhanced Recovery After Surgery (ERAS®) program [5], bed rest has no place in the treatment of acute diverticulitis.

### Antibiotics in Uncomplicated Diverticulitis

Routine antibiotic treatment of uncomplicated acute diverticulitis used to be, and in part still is, a standard practice. Several observational studies however show that omitting antibiotics may be safe. Two randomized clinical trials followed, published in 2012 and 2017 [6, 7•]. The Scandinavian AVOD trial [6] has randomized 623 CT-proven uncomplicated (Hinchey 1a only) diverticulitis patients, first or recurrent attack, to observational or antibiotic treatment. No differences are found in rates of complicated diverticulitis during initial hospital stay (1.9% (6/309) in the observational group versus 1.0% (3/314) in the antibiotic group; *p* = 0.302), emergency sigmoid resections (0.3% (1/309) in the observational group versus 1.0% (3/314) in the antibiotic group; *p* = 0.324), and recurrent diverticulitis at 1-year follow-up (16.2% (47/290) in the observational group versus 15.8% (46/292) in the antibiotic group; *p* = 0.881). The Dutch DIABOLO trial [7•] randomized 528 CT-proven uncomplicated (Hinchey 1a and 1b, see Table 1 for an explanation of the Hinchey classification) diverticulitis patients to observational or antibiotic treatment. The primary endpoint, time-to-recovery, as defined by fulfilling several clinical parameters such as discharge from hospital, normal diet, and low pain scores, was comparable among groups (median 14 days (IQR 6–35) in the observational group versus median 12 days (IQR 7–30) in the antibiotic group; *p* = 0.151). Also, secondary outcomes within 6 months of follow-up did not differ between groups; complicated diverticulitis (3.8% (10/262) versus 2.6% (7/266), respectively; *p* = 0.377), emergency surgery (0.8% (2/262) versus 1.1% (3/266), respectively; *p* = 0.553), overall morbidity (48.5% (127/262) versus 54.5% (145/266), respectively; *p* = 0.221), and recurrent diverticulitis (3.4% (9/252) versus 3.0% (8/266), respectively; *p* = 0.494. Observation alone, patients had a significantly shorter length of hospital stay (median 2 days (IQR 1–3) versus median 3 days (IQR 2–3) in the antibiotic group (*p* = 0.006). Results from these randomized clinical trials confirm that omitting antibiotics in the treatment of uncomplicated diverticulitis is without significant short-term and mid-term repercussions.

**Table 1:** Acute diverticulitis staging according to the modified Hinchey classification [8]

Stage	Definition
1a	Confined pericolic inflammation or phlegmon
1b	Pericolic or mesocolic abscess
2	Pelvic, distant intra-abdominal or retro-peritoneal abscess
3	Purulent peritonitis
4	Fecal peritonitis

### Outpatient Treatment

Until several years ago, routine intravenous antibiotic treatment made hospital admission inevitable. As at first oral antibiotic treatment and later treatment without antibiotics appeared to be safe in uncomplicated diverticulitis patients, outpatient treatment became feasible. Mainly in the last 5 years, several studies (four observational cohort studies and one randomized clinical trial) on outpatient treatment of imaging-proven acute diverticulitis were published. In observational studies, however, patients are assigned to inpatient or outpatient treatment based on disease severity and clinical condition hampering a reliable comparison of readmission and complication rates. Only two studies have made a fair comparison of inpatient versus outpatient treatment. One randomized clinical trial [9••] randomizing uncomplicated acute diverticulitis patients to inpatient or outpatient treatment, and a prospective cohort study [10] assigning uncomplicated diverticulitis patients to inpatient or outpatient treatment based on the time period before and after local protocol included outpatient treatment. The randomized clinical trial, including 132 patients, found no difference in readmission rate (4.5% (3/66) in the outpatient group versus 6.1% (4/66) in the inpatient group; *p* = 0.619) and no need for emergency surgery or percutaneous abscess drainage in either group [9••]. The prospective cohort study also found no difference in readmission rate (6.3% (2/32) in the outpatient group versus 0.0% (0/44) in the inpatient group; *p* = 0.174) and no need for emergency surgery or percutaneous abscess drainage in either group [10]. Both studies observed cost savings in favor of the outpatient group ranging from 67 to 82%. Most patients were readmitted because of persistent pain or vomiting in absence of any diverticular complications. Not all acute diverticulitis patients, however, are suited for outpatient treatment. Most studies only included patients with uncomplicated acute diverticulitis, without serious comorbidity or immunocompromised state, that were able to tolerate oral intake and had an adequate social or family network. For these types of patients, outpatient treatment seems to be safe.

### Pericolic Extraluminal Air

Along with an increasing usage and quality of computed tomography (CT) in diagnosing acute diverticulitis, pericolic extraluminal air is encountered more and more. Although in approximately 15% of all acute diverticulitis patients pericolic extraluminal air is seen, little is known about the natural course and whether these patients should be treated as uncomplicated diverticulitis or more aggressively as complicated diverticulitis. Nowadays, treatment of these patients is mainly based on the opinion and experiences of the physician, possibly causing over- or under treatment. Several studies including patients with acute diverticulitis and pericolic extraluminal air have been published so far, using a variety of terms like “free air within 5 cm of the inflamed colon segment,” “contained perforation,” “localized pericolic free air,” or “air within the mesentery” [11–16]. No more than one study evaluated the need for emergency surgery in isolated pericolic extraluminal air patients and in uncomplicated acute diverticulitis patients, but no events were observed in either group [14]. In six studies, 0 to 11% of patients needed emergency surgery within the initial acute diverticulitis episode [11–16]. Most of these rates are higher than the reported 1 to 2% need for emergency in uncomplicated diverticulitis in literature. Based upon this scarce evidence, an initial conservative approach is advocated in isolated pericolic extraluminal air patients.

## Patient Subgroups

### Young Patients

Large observational studies have suggested a more virulent disease course and higher recurrent diverticulitis rates in young patients. Most of these studies, however, are based on existing databases that lack an imaging-proven diagnosis of acute diverticulitis. This confirmation is essential to prevent that patients suffering from different diseases but with comparable symptoms, such as inflammatory bowel disease or irritable bowel syndrome, enter the study group. Studies that only include computed tomography-proven acute diverticulitis patients have found comparable proportions of complicated diverticulitis at presentation and comparable rates of emergency surgery in young (using cut-offs at 40 or 50 years) and elderly patients [17–20]. Nevertheless, these CT-based studies have found slightly higher rates of recurrent diverticulitis. However, one major limitation of these studies should be considered; these studies do not report the duration of follow-up for each age group separately, hampering the comparison of an outcome measure that relies mostly on the duration of follow-up in which a recurrence can occur [18, 20, 21]. In the only three studies that report the risk of recurrent diverticulitis using hazard ratios, in which the follow-up duration of each patient is taken into account, comparable risks of recurrent diverticulitis are found for young and elderly patients [22, 23••, 24]. In summary, young patients do not suffer from a more virulent or recurrent disease course compared to elderly patients. Studies on the risk of recurrent diverticulitis show conflicting results, but those studies with the most reliably design have not found an association with age. Consequently, young patients should not be treated more aggressively, in particular surgically, for the prevention of complications or recurrences.

### Immunocompromised Patients

The role of immunosuppression in the natural course of acute diverticulitis is studied most reliably in post-transplant patients. In these studies, treatment is well documented and the immunosuppressed state of patients is a fact. Other immunosuppressed patient groups have also been studied, such as patients using steroids and diabetic patients. A systematic review, including 17 observational studies and a total of 11,866 post-transplant patients, demonstrates that a pooled 40% (95% CI 32–50%) of post-transplant patients with acute diverticulitis have complicated diverticulitis [25]. Two observational studies that were published more recently reported even higher proportions of complicated diverticulitis in post-transplant patients; 57 and 56% [26, 27]. In non-transplant patients, approximately one-third of all acute diverticulitis patients have complicated disease.

A systematic review of five studies comparing patients with and without steroid use shows significantly higher odds of diverticular perforation in patients on steroids (odds ratio 9.1; 95% CI 3.5–23.6) [28]. The association between immunosuppression and complicated diverticulitis is less obvious in patients with diabetes mellitus. Two observational studies report proportions of patients with complicated diverticulitis in diabetic and non-diabetic patients and show opposite results [29, 30]. The one study [29] reports a significantly higher proportion of complicated diverticulitis in diabetic patients (44 versus 32%, *p* = 0.005), while the other study [30] reports a non-significantly lower rate (19 versus 25%, *p* = 0.457) and a significantly lower adjusted odds ratio 0.18 (95% CI 0.04–0.80) for complicated disease in diabetic patients. In summary, immunosuppressive medication is associated with a higher risk of complicated acute diverticulitis. This association has not been demonstrated in diabetic patients.

### Patients on Medications

Several types of medication have been linked to a more complicated or less complicated course of acute diverticulitis. For some types of medication, a possible underlying mechanism is suggested. Non-steroidal anti-inflammatory drugs (NSAIDs) may affect inflammation and increase the rate of complicated diverticulitis. Calcium channel antagonists may reduce the rate of complicated diverticulitis due to their relaxation of smooth muscle.

A systematic review including ten case control studies and one cohort study assessed NSAIDs, aspirin, opioids, and calcium channel antagonists as potential risk factors for complicated diverticulitis [28]. All 11 included studies found a higher risk (and in ten of these, a significantly higher risk) of complicated diverticulitis in previous or current NSAID users. The pooled odds ratio of complicated diverticulitis in NSAID users is 2.49 (95% CI 1.98–3.14) compared to control groups. Also, patients on opioids appear to be at higher risk of developing complicated diverticulitis. Pooled results from three studies yield an odds ratio of 2.52 (95% CI 1.77–3.57). For both NSAIDs and opioids, it is relevant to know whether cessation or start of these drugs at the time of the acute diverticulitis diagnosis influences the natural course of diverticulitis, but this question remains unanswered thus far. Neither aspirin nor calcium channel antagonists are associated with a higher risk of complicated diverticulitis (pooled odds ratio 1.03 (95% CI 0.69–1.55) and pooled odds ratio 0.70 (95% CI 0.37–1.34), respectively).

## Prevention of Recurrent Diverticulitis

Cyclic or continuous pharmacological therapies to prevent recurrent diverticulitis after an acute episode have been subject of research. The main candidates are rifaximin (a poorly absorbed antibiotic), mesalazine (an anti-inflammatory agent), and probiotics (live microorganisms that may alter or restore the gut microbiome). Although these therapies were frequently studied in patients with asymptomatic diverticulosis or symptomatic diverticular disease, studies in imaging-proven acute diverticulitis patients are scarce. Pharmacological effects in patients with symptomatic diverticular disease may well differ from those in patients with acute diverticulitis. Therefore, to study the effect of pharmacological therapies on acute diverticulitis patients, an imaging-proven diagnosis is needed.

### Antibiotics (Rifaximin)

Two observational cohort studies on rifaximin treatment of imaging -proven acute diverticulitis patients have been published [31, 32]. Both studies compare rifaximin treatment with mesalazine treatment instead of comparison with placebo or no treatment at all. One was a retrospective study including 124 patients and showed that the risk of recurrence was lower in the rifaximin group compared with the mesalazine group (hazard ratio adjusted for age and gender 0.27; 95% CI 0.10–0.72). The other study was a prospective cohort study that demonstrated opposite results. In the rifaximin group (treated 7 days a month), significantly, more patients developed recurrent diverticulitis compared with daily mesalazine treatment. Importantly, this study reports only *p* values and fails to report any raw data.

Currently, there is insufficient evidence that rifaximin prevents recurrent diverticulitis.

### Anti-Inflammatory Drugs (Mesalazine/5-ASA)

Three studies, reporting results from four randomized, placebo-controlled trials, have been published. An Italian study [33] randomized patients to a monthly 10-day cycle of mesalazine or placebo treatment. Recurrent diverticulitis rates were comparable at 12 months (RR 0.88; 95% CI 0.29–2.71), and slightly but not significantly lower in the mesalazine group at 24 months (RR 0.49; 95% CI 0.20–1.19). An American study [34] randomized patients to daily mesalazine or placebo treatment during 3 months and followed patients for 12 months. Rates of recurrent diverticulitis were comparable in this study (RR 0.91; 95% CI 0.42–1.97). Another two randomized, placebo-controlled trials have been published in one paper recently [35]. Both trials were multinational studies, taking place in 11 and nine countries, respectively, randomizing patients to daily mesalazine or placebo treatment during the entire follow-up duration. In both trials, no differences were found in the proportion of patients free of recurrent diverticulitis at 48 weeks; 67.9% in the mesalazine group versus 74.4% in the placebo group (*p* = 0.226) in one trial, and 52.0% in the mesalazine group versus 58.0% in the placebo group (*p* = 0.860) in the other trial. Also after 96 weeks of treatment and follow-up in one of the trials, no differences were found. In summary, although effects on complaints in patients with symptomatic diverticular disease have been found [36], the four randomized placebo-controlled trials in patients with imaging proven acute diverticulitis show no effect of mesalazine in the prevention of recurrent diverticulitis.

### Probiotics

Only one study, a randomized controlled trial, assessed the efficacy of probiotics in imaging proven acute diverticulitis patients [34]. Patients were randomized to mesalazine monotherapy, combined mesalazine and probiotic treatment, or placebo treatment for 3 months. At a follow-up duration of 12 months, the three study groups showed comparable results; 31% of patients in the combined mesalazine and probiotics group developed recurrent diverticulitis, versus 31% of the placebo group and 28% of the mesalazine group. Also, no statistically significant differences were found for the amount and severity of symptoms at 3 and 12 months. No study compared probiotic monotherapy with placebo treatment. Further studies are needed to draw firm conclusions but as yet, the available evidence does not support the use of probiotic treatment for prevention of recurrent diverticulitis.

## Conclusions and Future Perspectives

To study the effects of treatment in acute diverticulitis patients, an imaging proven diagnosis of acute diverticulitis is essential. Studies without this diagnostic confirmation include patients that suffer with other diseases such as symptomatic uncomplicated diverticular disease, irritable bowel syndrome, or inflammatory bowel disease. Results from these types of studies cannot be reliably extrapolated to acute diverticulitis patients. Therefore, this review has focussed on the available evidence on conservative treatment of imaging proven acute diverticulitis. Table 2 shows the summary of available evidence.

**Table 2:** Summary of current evidence on the conservative treatment of acute diverticulitis

	Intervention or group	Conclusion	Evidence
Treatment strategies in acute diverticulitis	Diet	Unrestricted diet seems justified	Some low quality and observational evidence shows the safety of an unrestricted diet, whereas no evidence in favor of dietary restrictions exists.
	Bed rest	No place for bed rest in the treatment of acute diverticulitis	Beneficial effects have never been studied nor proven.
	Antibiotics in uncomplicated diverticulitis	Omitting antibiotics is safe in uncomplicated diverticulitis patients.	Two RCT’s show that omitting antibiotics is without significant short-term and mid-term repercussions.
	Outpatient treatment	Safe for uncomplicated diverticulitis patients without serious comorbidity or immunocompromised state and with an adequate social network	One RCT and an observational study show no increased readmission rate. Readmissions are predominantly because of vomiting or persistent pain instead of diverticular complications.
	Pericolic extraluminal air	Initial conservative approach is advocated in isolated pericolic extraluminal air patients.	Although slightly higher than for uncomplicated diverticulitis patients in literature, rates of need for emergency surgery are relatively low in observational studies.
Altered disease course in patient subgroups	Young patients	No more virulent or recurrent disease course compared to elderly patients	Observational studies show comparable proportions of complicated diverticulitis. Studies that take follow-up duration into account, have not found an association between recurrent diverticulitis and age.
	Immuno-compromised patients	Immunosuppressive medication is associated with higher risk of complicated diverticulitis; diabetes mellitus is not.	Observational studies show higher risks of complicated diverticulitis in post-transplant patients or patients on steroids. Studies with diabetic patients report conflicting results.
	Patients with medication	Patients on NSAIDs or opioids are at higher risk of complicated diverticulitis.	Mainly case-control studies show higher risks of complicated diverticulitis in patients on NSAIDs or opioids. The effect of start or cessation of these drugs at the time of diverticulitis presentation has not been studied.
Pharmacological prevention of recurrent diverticulitis	Rifaximin	Insufficient evidence to conclude on efficacy	Two observational studies comparing rifaximin with mesalazine show conflicting results.
	Mesalazine	No beneficial effect on preventing recurrent diverticulitis	Four placebo-controlled RCTs show no differences in rates of recurrent diverticulitis between groups.
	Probiotics	Insufficient evidence to conclude on efficacy	One RCT comparing placebo with combined mesalazine and probiotics treatment shows no difference in recurrence rates.

Several treatment strategies that previously have been imposed as routine treatment are now obsolete. Dietary restrictions and bed rest have no place in the treatment of acute diverticulitis any more. Omitting antibiotics for uncomplicated diverticulitis has shown to be safe, and therefore antibiotics should not be used in these patients routinely. This recent development has opened doors for outpatient treatment, as inpatient treatment with antibiotics is no longer needed. Outpatient treatment is safe in uncomplicated diverticulitis patients without serious comorbidity or immunosuppression, who are able to tolerate oral intake and have an adequate social network. Patients with pericolic extraluminal air on imaging are seen more frequently because of the increasing use of computed tomography. Conservative treatment has shown to be successful in the vast majority of these patients. Patients on immunosuppressive and non-steroidal anti-inflammatory drugs seem to have a higher risk of developing complicated diverticulitis. Young patients are not at risk for a more virulent disease course. Data regarding risk of recurrent diverticulitis in young patients seem conflicting but when the follow-up duration is taken into account, rates of recurrent diverticulitis are comparable to rates in elderly patients. To date, no pharmacological therapies have been able to prevent recurrence of diverticulitis after an acute episode. Mesalazine did not show any effect in four randomized placebo-controlled trials, whereas rifaximin and probiotics have not been studied sufficiently to conclude anything on their efficacy. Although high fiber diets or some form of dietary restriction are frequently recommended following an episode of acute diverticulitis, these strategies have not been proven. Even data on efficacy of dietary restrictions or supplementation in symptomatic diverticular disease patients is scarce, inconsistent, and of very low quality. For the prevention of recurrent disease in imaging-proven acute diverticulitis patients, there is a complete lack of evidence for efficacy of dietary interventions. As a consequence, there are no grounds for dietary advice to prevent recurrent episodes or complaints after acute diverticulitis.

For patients with acute diverticulitis, a great deal is to be gained when evidence-based treatment is implemented in daily practice, instead of treating patients based on traditional understandings or inadequate interpretation of available evidence. A topic that urgently needs be addressed in future research is the pharmacological prevention of recurrent diverticulitis and persistent complaints. Furthermore, future studies should focus on the identification of patients at risk for the development of complications or persisting complaints from acute diverticulitis in order to develop patient-tailored treatment strategies, either by new treatments or by using more aggressive strategies in well selected patients with initially uncomplicated diverticulitis.
